# 53/m mit neudiagnostiziertem Leberherd bei vorbekannter Leberzirrhose: Vorbereitung auf die Facharztprüfung: Fall 2

**DOI:** 10.1007/s00104-022-01701-z

**Published:** 2022-08-29

**Authors:** A. Ali Deeb, U. Settmacher

**Affiliations:** Klinik für Allgemein‑, Viszeral- und Gefäßchirurgie, Universitätsklinikum Jena, Am Klinikum 1, 07747 Jena, Deutschland

**Keywords:** Heptozelluläres Karzinom, Model for End-Stage Liver Disease

## Prüfungssimulation

### Fallschilderung

Die Vorstellung des Patienten erfolgt im Leberzentrum aufgrund zuletzt im letzten halben Jahr mehrfacher, hydropischer, hepatischer Dekompensationen bei seit 7 Jahren vorbekannter, histologisch gesicherter äthyltoxischer Leberzirrhose im Stadium Child-Pugh B. Davor sei der Patient deswegen selten beim Arzt gewesen bzw. im Krankenhaus aufgenommen worden. Er war aber regelmäßig zur jährlichen Routinekontrolle beim Hausarzt. Bezüglich des Alkoholkonsums gibt der Patient glaubhaft eine strikte Alkoholkarenz seit der Erstdiagnose der Leberzirrhose an. Zum Zeitpunkt der Vorstellung befand sich der Patienten in einem kompensierten Allgemein- und sarkopenischen Ernährungszustand. Die hepatische Funktion war klinisch sowie laborchemisch kompensiert. Der Lab-MELD(Model for End-Stage Liver Disease)-Score betrug 14. Bei der klinischen Untersuchung besteht eine erstgradige hepatische Enzephalopathie. Der Ballotement-Test ist bei mäßigem Aszites positive. Im Rahmen der Vorstellung wird eine Lebersonographie durchgeführt. Hierbei fällt ein suspekter Leberherd im Segment VIII mit einem Durchmesser von ca. 3 cm auf. Der Tumormarker AFP (Alpha-Fetoprotein) lag mit 2,8 ng/ml im Normbereich.

## Prüfungsfragen

- Wie lautet Ihre erste Verdachtsdiagnose?
- Was würden Sie hinsichtlich der Vorsorge als kritisch betrachten?
- Anhand welcher Verfahren und Befunde kann die suspekte Läsion in der Bildgebung weiter abgeklärt werden?
- Welche Therapieoption würden Sie dem Patienten anbieten?
- Welche Maßnahmen würden Sie in der Zeit bis zur Transplantation in Angriff nehmen?
- Wie geht es nach der Lebertransplantation weiter? Würden Sie eine adjuvante Therapie empfehlen?
- Im Falle von Kontraindikationen für eine Lebertransplantation, welche Therapieoptionen würden Sie empfehlen? Nennen und diskutieren Sie mindestens drei Therapieoptionen.

### Antworten

#### Wie lautet Ihre erste Verdachtsdiagnose?

Bei einem unklaren Leberherd auf dem Boden einer Zirrhose jeglicher Ursache soll das hepatozelluläre Karzinom als erste Verdachtsdiagnose abgeklärt werden.

#### Was würden Sie hinsichtlich der Vorsorge als kritisch betrachten?

Die Inzidenz maligner Lebertumoren ist in den letzten Dekaden deutlich angestiegen. Das primäre Leberzellkarzinom (HCC) ist hierbei der weltweit häufigste maligne Lebertumor. Eine Leberzirrhose ist der wichtigste Risikofaktor für die Entwicklung eines HCC unabhängig von der zugrunde liegenden Ätiologie. Daher soll Patienten mit Leberzirrhose eine regelmäßige Diagnostik zur HCC-Früherkennung angeboten werden. Die Teilnahme an dieser kann darüber hinaus den Patienten mit fortgeschrittener Leberfibrose angeboten werden [1]. Dafür soll alle 6 Monate eine Ultraschalluntersuchung der Leber durchgeführt werden. Eine AFP-Bestimmung kann auch durchgeführt werden.

Im aktuellen Kasus wurde dem Patienten keine derartige Früherkennung angeboten, obwohl die Diagnose einer Leberzirrhose seit längerer Zeit gesichert war. Dieser Punkt soll kritisch diskutiert werden.

##### Merke.

Patienten mit Leberzirrhose soll eine HCC-Früherkennung (halbjährlich) mittels Sonographie und ggf. AFP angeboten werden.

#### Anhand welcher Verfahren und Befunde kann die suspekte Läsion in der Bildgebung weiter abgeklärt werden?

- Das HCC soll primär anhand seiner typischen Kontrastmitteldynamik mit arterieller Hypervaskularisation und Auswaschen in der portalvenösen und venösen Phase in der kontrastverstärkten Magnetresonanztomographie (MRT) bei Raumforderungen mit einem Durchmesser von > 1 cm diagnostiziert werden. Eine zweite kontrastverstärkte Bildgebung (Computertomographie [CT] bzw. Sonographie) ist nur dann notwendig, wenn kein charakteristisches Kontrastmittelverhalten im MRT zu sehen ist. Dies ist für die Diagnosestellung mit kurativer Therapieintention ausreichend, dafür ist keine histologische Sicherung erforderlich [1].
- Unklare Leberherde < 1 cm sollten in 3‑Monats-Intervallen mit dem bestgeeigneten kontrastmittelverstärkten Schnittbildverfahren kontrolliert werden.
- Zum Tumorstaging ist eine ergänzende Computertomographie des Thorax erforderlich.

**Figure 1:**
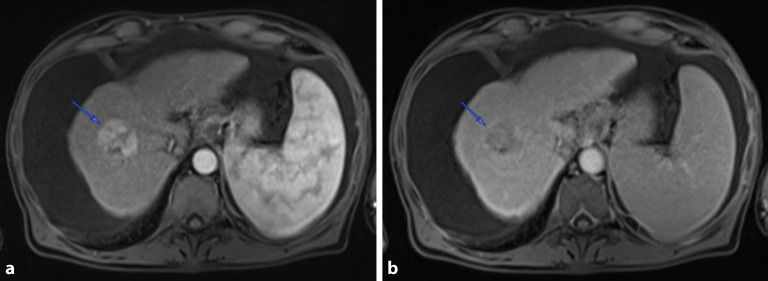

Im aktuellen Kasus ergaben die MRT- und CT-Untersuchungen die folgenden Ergebnisse (Abb. 1):

- inhomogene Läsion in den Lebersegmenten VII/VIII bis maximal 4 cm im Durchmesser, mit Hypervaskularisation arteriell und Auswaschphänomen portalvenös, somit auf HCC bei Leberzirrhose verdächtig; ansonsten keine weiteren suspekten Herde,
- keine Makrogefäßinvasion,
- keine extrahepatische Manifestationen nachweisbar,
- ausgeprägter Aszites.

##### Merke.

Im Falle einer kurativen Therapieintention ist keine histologische Sicherung bei Leberherden mit typischem Kontrastmittelverhalten erforderlich.

#### Welche Therapieoption würden Sie dem Patienten anbieten?

- Der solitäre Befund in unserem Fall ist zwar onkologisch, aber nicht funktionell resektabel. Es besteht eine Child-B-Zirrhose mit Aszites und einem MELD von 14. Aufgrund der erheblichen postoperativen Morbidität und Mortalität ist bei dieser Funktionseinschränkung von der Resektion abzusehen.
- Die lokal-ablativen Verfahren sind aufgrund des relativ hohen Rezidivrisikos bei Herden > 3 cm alleinig nicht die Therapie der ersten Wahl.
- Prinzipiell besteht für die meisten Patienten mit einem auf die Leber begrenzten HCC eine hohe Chance auf eine kurative Behandlung durch eine Lebertransplantation. Wichtig für die Auswahl geeigneter Patienten ist der zu erwartende Vorteil für die Transplantation im Vergleich zu alternativen Behandlungsstrategien. Daneben spielt auch die beschränkte Verfügbarkeit von Spenderorganen eine Rolle. Neben der onkologischen Prognose muss die allgemeine Lebenserwartung und das Alter des Patienten in die Entscheidungsfindung einbezogen werden. Aus Mangel an etablierten tumorbiologischen Kriterien (mit Ausnahme des Differenzierungsgrades und des AFP-Werts, s. unten), orientieren sich die verfügbaren Analysen vor allem an morphometrischen Kriterien, d. h. an der Anzahl und Größe der Tumorknoten. Bei rein hepatischem Befall korreliert das Rezidivrisiko nach Lebertransplantation mit der Anzahl und (maximalen bzw. kumulativen) Größe der vorhandenen Tumorknoten. Patienten mit morphologisch kleiner Tumorlast haben eine besonders günstige Prognose nach Lebertransplantation. So hatten Patienten mit einem irresektablen HCC innerhalb der Mailand-Kriterien (Tab. 1) nach Lebertransplantation eine 5‑Jahres-Überlebensrate von 65–80 % und eine HCC-Rezidivrate von etwa 10–12 %. Daher stellen die Mailand-Kriterien auch in Deutschland die akzeptierte Basis für eine Priorisierung dieser Patienten zur Lebertransplantation dar.

**Table 1:** 

	Solitärer Knoten	Mehrere Knoten
Keine extrahepatischen Manifestationen (cN0 cM0)		
Keine makrovaskuläre Gefäßinvasion (cV0)		
Mailand-Kriterien	≤ 5 cm	Max 3 Herde ≤ 3 cm
BÄK-Richtlinien	(BÄK ≥ 1 cm und ≤ 5 cm)	(BÄK ≥ 2 cm und ≤ 3 cm)

##### Der Fall.

In unserem Kasus handelt es sich um einen solitären Herdbefund auf dem Boden einer Leberzirrhose mit maximalem Durchmesser von 4 cm. Somit liegt der Befund innerhalb der Mailand-Kriterien sowie innerhalb der BÄK(Bundesärztekammer)-Richtlinien. Daher stellt die Lebertransplantation die Therapie der ersten Wahl bei am besten zu erwartendem Gesamt- und rezidivfreien Überleben dar. Der Patient kann auch bei der Listung zur Lebertransplantation priorisiert werden (Beantragung einer „standard exception“). Es bestehen keine Kontraindikationen für die Lebertransplantation. Der Patient wird evaluiert und auf die Warteliste für eine Lebertransplantation aufgenommen.

#### Welche Maßnahmen würden Sie in der Zeit bis zur Transplantation in Angriff nehmen?

- Patienten auf der Warteliste haben ein relevantes Risiko für einen Tumorprogress und somit für eine eventuelle Abmeldung von der Warteliste. Dieses Risiko kann man durch eine Bridging- bzw. Downstaging-Therapie senken.
- Hierfür sollen lokal-ablative Verfahren, Resektionen oder transarterielle Verfahren (transarterielle Chemoembolisation [TACE], selektive interne Radiotherapie [SIRT]) eingesetzt werden. Eine Hochpräzisionsradiotherapie („stereotactic body radiotherapy“, SBRT) kann als Bridging-Verfahren bis zur Lebertransplantation ebenfalls erwogen werden, wenn die weiteren Verfahren nicht zum Einsatz kommen können. Die Auswahl des geeigneten Verfahrens soll interdisziplinär in einem Tumorboard eines Transplantationszentrums getroffen werden. Die Bridging‑/Downstaging-Therapie kann nach Bedarf im Falle der Restvitalität des Tumors wiederholt werden.
- Eine vierteljährliche bildgebende Verlaufskontrolle mit der bestgeeigneten Diagnostik soll erfolgen, um einen eventuellen Tumorprogress, und eine eventuell damit verbundene Kontraindikation zur Lebertransplantation, auszuschließen.

##### Der Fall.

In unserem Kasus war der solitäre Befund für eine transarterielle Chemoembolisation gut zugänglich. Von einer Resektion bzw. Ablation war bei nicht sicher ausreichender Restfunktion abzusehen. Daher wurde im Konsens des interdisziplinären Tumorboards für eine TACE entschieden. Diese erfolgte komplikationslos. Die 6 Wochen danach durchgeführte CT-Verlaufskontrolle ergab keine Restvitalität des Leberherdes. Des Weiteren zeigte sich ansonsten keine Befunddynamik. Der Befund blieb für ca. 9 Monate stabil, bis ein geeignetes Organangebot vorlag und die Lebertransplantation daraufhin durchgeführt werden konnte. Histologisch ergab sich ein hepatozelluläres Karzinom mit der Tumorformel: ypT1b pN0 M0, L0 V0 Pn0, UICC(Union Internationale Contre le Cancer)-Stadium IB, R0, G entfällt nach TACE.

##### Merke.

Bridging-Therapie: lokoregionäre Therapie oder Resektion eines HCC innerhalb der Mailand-Kriterien auf der Warteliste.

#### Wie geht es nach der Lebertransplantation weiter? Würden Sie eine adjuvante Therapie empfehlen?

- Für eine adjuvante Tumortherapie gibt es bisher keine evidenzbasierte Grundlage, dass das Rezidivrisiko dadurch verringert werden kann. Daher sollen Patienten mit HCC nach Lebertransplantation außerhalb von Studien nicht adjuvant behandelt werden.
- Die Nachsorge nach erfolgreicher Therapie soll im ersten Jahr alle 3 Monate und im zweiten Jahr alle 6 Monate über insgesamt 5 Jahre mittels biphasischer kontrastverstärkter CT oder MRT stattfinden.

#### Im Falle von Kontraindikationen für eine Lebertransplantation, welche Therapieoptionen würden Sie empfehlen? Nennen und diskutieren Sie mindestens drei Therapieoptionen.

Aufgrund mehrerer möglicher Therapiemodalitäten und aufgrund unterschiedlicher Ausprägung der Leberzirrhose sollen Patienten mit einem HCC in einer interdisziplinären Tumorkonferenz vorgestellt werden. Folgende Therapieoptionen außerhalb der Lebertransplantation könnten angeboten werden:

- **Resektion:** Bei der Beurteilung der funktionellen Resektabilität nehmen die Einschätzung der Leberfunktion und des Ausmaßes der portalen Hypertension eine zentrale Rolle ein. Besonders bei Patienten mit Leberparenchymstörungen sollte die Leberfunktionsreserve beurteilt und das Risiko der Entwicklung eines postoperativen Leberversagens eingeschätzt werden. Hierzu haben sich neben der klassischen Child-Pugh-Stadieneinteilung unter anderem der Indocyaningrün-Clearance-Test und der LiMAx(„maximum liver function capacity“)-Atemtest sowie das Leberelastogramm bewährt. Der ideale Patient für eine Resektion mit einem HCC in Zirrhose hat einen kleinen einzelnen HCC-Knoten in peripherer Lage und eine gut erhaltene Leberfunktion mit einer Thrombozytenzahl >100.000/ml. Allerdings kann eine Leberresektion auch bei multiplen HCC-Knoten in Leberzirrhose durchgeführt werden, sofern diese technisch durchführbar ist und ausreichend funktionelles Lebergewebe erhalten werden kann [1].
- **Lokale Ablation:** Bei Patienten mit HCC bis 3 cm sind die Resektion und die Ablation nahezu äquivalente Verfahren. Daher soll bei diesen Patienten mit HCC kleiner als 3 cm in für die Resektion ungünstiger Lokalisation oder mit eingeschränkter Leberfunktion primär eine Thermoablation des Tumors angeboten werden. Die Ablation des HCC soll mittels Radiofrequenzablation (RFA) oder Mikrowellenablation (MWA) über einen perkutanen, laparoskopischen oder offenen Zugang durchgeführt werden [1].
- **Transarterielle Chemoembolisation (TACE):** Die TACE ist indiziert bei Patienten mit multinodulärem oder großem HCC, wenn keine potenziell kurativen Therapieoptionen vorliegen und nach Ausschluss folgender Kontraindikationen [1]:
  - fortgeschrittenes Erkrankungsstadium mit tumorbedingten Symptomen und Reduktion des Allgemeinzustandes (ECOG [Eastern Cooperative Oncology Group] ≥2),
  - dekompensierte Lebererkrankung (Child-Pugh C) oder hohe Tumorlast und reduzierte Leberfunktion (Child-Pugh B >7),
  - gesicherte prognoserelevante extrahepatische Metastasierung,
  - komplette Pfortaderthrombose oder komplette hepatofugale Pfortaderperfusion,
  - hypovaskularisiertes HCC in CT oder MRT.
- **Transarterielle Radioembolisation (TARE):** Die TARE kann nach Beschluss des Tumorboards bei Patienten mit erhaltener Leberfunktion im intermediären HCC-Stadium anstelle einer TACE eingesetzt werden. Prinzipiell gibt es aktuell einige Studien, die eine Vergleichbarkeit der TARE mit der TACE belegen, jedoch keine Überlegenheit eines der beiden Verfahren herausarbeiten konnten. Allerdings belegen mehrere Studien die Wirksamkeit sowie die Sicherheit der TARE bei Patienten mit Pfortaderthrombosen [1].
- **Stereotaxie:** Eine Hochpräzisionsradiotherapie („stereotactic body radiotherapy“, SBRT) kann in Betracht gezogen werden, wenn andere lokale Therapieverfahren nicht möglich sind (z. B. hohe Wahrscheinlichkeit für ein Therapieversagen, eingeschränkte Leberfunktion, technische Hindernisse) [1].
- **Systemtherapie:** Für HCC-Patienten mit erhaltener Leberfunktion (im Child-Pugh-Stadium A), mit Fernmetastasen und/oder einer Tumorlokalisation, die lokoregionär nicht kontrolliert oder reseziert werden kann, soll eine Systemtherapie angeboten werden. Dabei soll eine Kombination von Atezolizumab und Bevacizumab (A + B) angeboten werden. Bei Kontraindikation für A + B soll eine Therapie mit einem der beiden Tyrosinkinaseinhibitoren Lenvatinib oder Sorafenib angeboten werden [1].
